# Lifestyle practices that reduce seasonal PM_2.5_ exposure and their impact on COPD

**DOI:** 10.1038/s41598-023-38714-5

**Published:** 2023-07-21

**Authors:** Hajeong Kim, Jin-Young Huh, Geunjoo Na, Shinhee Park, Seung Won Ra, Sung-Yoon Kang, Ho Cheol Kim, Hwan-Cheol Kim, Sei Won Lee

**Affiliations:** 1 Department of Pulmonary and Critical Care Medicine, University of Ulsan College of Medicine, Asan Medical Center, 88 Olympic-ro 43–gil, Songpa-gu, Seoul, 05505 Republic of Korea; 2 Division of Pulmonary, Allergy and Critical Care Medicine, Department of Internal Medicine, Hallym University Kangdong Sacred Heart Hospital, Seoul, Republic of Korea; 3 Division of Pulmonary, Allergy and Critical Care Medicine, Department of Internal Medicine, Chung-Ang University Gwangmyeong Hospital, Chung-Ang University College of Medicine, Gwangmyeong, Republic of Korea; 4 Department of Occupational and Environmental Medicine, College of Medicine, Inha University, 27 Inhang-Ro, Jung-gu, Incheon, 22332 Republic of Korea; 5 Green Environment Industrial Institute, Seoul, Republic of Korea; 6 Department of Pulmonary, Allergy and Critical Care Medicine, Gangneung Asan Hospital, Gangneung, Republic of Korea; 7 Division of Allergy and Respiratory Medicine, Department of Internal Medicine, Soonchunhyang University Bucheon Hospital, Bucheon, South Korea; 8 Division of Pulmonary and Critical Care Medicine, Department of Internal Medicine, Ulsan University Hospital, University of Ulsan College of Medicine, Ulsan, Republic of Korea; 9 Department of Internal Medicine, Gachon University Gil Medical Center, Incheon, Republic of Korea

**Keywords:** Environmental sciences, Health care, Medical research

## Abstract

Particulate matter (PM) is a major air pollutant that has led to global health concerns and can cause and exacerbate chronic obstructive pulmonary disease (COPD). We asked patients with COPD to complete a detailed questionnaire about their lifestyle practices to reduce PM_2.5_ exposure and analyzed the relationship between ambient PM_2.5_ concentrations and lifestyle practices. We prospectively enrolled 104 COPD patients from four hospitals in different areas of Korea. They completed detailed questionnaires twice (at enrollment and the end of the study) and Internet of Things-based sensors were installed in their homes to continuously measure PM_2.5_ for 1 year. The relationship between PM_2.5_ concentrations, lifestyle practices, and COPD exacerbations were analyzed in each season. The PM_2.5_ concentration was higher outdoors than indoors in all seasons except summer, and the difference was largest in winter. The six lifestyle practices that significantly lowered the annual indoor PM_2.5_ concentration compared with the outdoors. The higher the economic status and educational level of patients, the lower the indoor PM_2.5_ concentration. Some lifestyle practices were associated with reduced small airway resistance, presented as R5–R20 determined by impulse oscillometry, and scores of the St. George’s Respiratory Questionnaire. Some lifestyle practices are associated with reduced indoor PM_2.5_ concentrations and can even affect clinical outcomes, including small airway resistance and quality of life of COPD patients.

## Introduction

Air pollution is a major public health threat and is estimated to cause 7 million deaths worldwide each year^1–3^. Due to nationwide efforts of some developed countries, air quality has improved and beneficial effects have been reported^4^. However, the concentrations of air pollutants far exceed the upper limit defined by the World Health Organization in developing countries^2^. Moreover, socially vulnerable people are also vulnerable to various air pollutants even in developed countries^5^. There is no lower limit of air pollution that does not affect our health^6^. These findings suggest that the lower exposure to air pollution, the better our health, and great efforts should be made to reduce exposure to air pollution.

Identification and control of pollution sources is the best approach to improve air pollution, but this is not easily achieved in most countries. It is particularly complicated to analyze pollution sources in Korea, which is located between Asia, China, and the Pacific Ocean. Due to its location, PM concentrations dynamically change over seasons due to fluctuations in pollutants from domestic and foreign sources according to alterations in the wind direction^7^. Countries with transboundary air pollution cannot improve air quality solely through domestic regulation. No specific system has been established to share information across jurisdictions between countries in Northeast Asia^8^, indicating it will be difficult to dramatically reduce ambient pollutants in the near future and that individual efforts to reduce exposure are required.

Patients with chronic respiratory diseases are susceptible to air pollutants. PM with a diameter smaller than 2.5 µm (PM_2.5_) is associated with hospitalizations and mortality of patients with chronic obstructive pulmonary disease (COPD)^9,10^ and is even considered a risk factor for this disease^11^. COPD is neither fully reversible nor medically curable and is a major cause of human mortality. Reduction of outdoor ambient PM_2.5_ can be advantageous for patients with COPD and can be achieved by nationwide efforts and international collaborations^12^, but is not easily accomplished as mentioned earlier. A similarly large number of deaths is associated with exposure to indoor air pollution, which can be controlled by individuals, and it takes less time to reduce air pollution indoors than outdoors. In this context, guidance to effectively reduce indoor PM_2.5_ exposure must be developed. However, most lifestyle guidelines to avoid PM_2.5_ exposure are based on experts’ opinions without definite evidence. We hypothesized that indoor PM_2.5_ concentrations are affected by lifestyle behaviors and can be reduced by appropriate lifestyle modifications. To develop an effective strategy and guide patients, we performed a detailed questionnaire survey about daily habits to reduce PM exposure of patients with COPD and measured real-time PM_2.5_ concentrations in their homes for 1 year. By analyzing lifestyle practices and indoor and outdoor PM_2.5_ concentrations according to the season in patients with COPD, we aimed to elucidate lifestyle behaviors that improve indoor PM_2.5_ concentrations and to determine the impact of PM_2.5_ concentrations on acute COPD exacerbations.

## Materials and methods

### Participants

This prospective panel study recruited patients with COPD from four representative areas of Korea: two metropolitan areas (Seoul, Asan Medical Center and Incheon, Gachon University Gil Medical Center), an industrialized area (Ulsan, Ulsan University Hospital), and a clean rural area (Gangwon province, Gangneung Asan Hospital). The inclusion criteria were (1) adults aged 40 years or older; (2) diagnosis of COPD, defined as post-bronchodilator forced expiratory volume in 1 s (FEV_1_)/forced vital capacity (FVC) < 0.7; and (3) predicted FEV_1_ less than 80% of the predicted value at enrollment. The exclusion criteria were (1) patients without respiratory symptoms and (2) patients who could not understand the questionnaires used in the study or instructions about how to use the air sampler device. This study was approved by the Institutional Review Boards of Asan Medical Center (2019-0476), Ulsan University Hospital (2019–07–049), Gangneung Asan Hospital (2019-06-049), and Gil Medical Center (GBirb2019-290). The detailed study design was published previously^13^.

### Study design

Demographic and clinical data, including data about age, sex, current address, concurrent asthma, and history of smoking, were collected at enrollment after obtaining written informed consent. Detailed questionnaire surveys were completed by the participants. Internet of Things (IoT)-based sensors were installed in their homes to measure indoor PM_2.5_ concentrations. The presence of COPD exacerbations was checked every month. Indoor PM_2.5_ concentrations were continuously monitored for 1 year. The associations between indoor PM_2.5_ concentrations and responses to the questionnaires and their impact on COPD exacerbations were analyzed.

### Questionnaires about lifestyles, social environment, and clinical data collection

Participants completed questionnaires about their indoor and outdoor environments and lifestyle practices to avoid PM exposure. Questions about the indoor environment asked about the method of indoor ventilation, presence of an indoor ventilating system, whether the kitchen and living room were separated, and use of household appliances such as air filters. Questions about the outdoor environment asked about the distance of their home from the road and traffic volume. The questionnaire about lifestyle practices included 20 practice items for which the response scale ranged from score 0 (have never practiced) to score 7 (practiced every day, Table S1). The questionnaire was formed based on a list of recommended behaviors from our national health department^14–16^ and a list of protective interventions from a literature review^17^. All questionnaires were completed twice, at enrollment and the end of the study (1 year). Information about educational level and economic status was also collected^13^. Patients attending Asan Medical Center underwent serial impulse oscillometry. All institutions the subjects’ information was sourced from obtained the informed consent. The data used encrypted identification of the subjects, thus written consent was not required from the patients. All procedures and methods were conducted in accordance Declaration of Helsinki.

### Measurements of PM exposure

Indoor PM_2.5_ concentrations were measured using a sensor-based light scattering measurement device (CP-16-A5; Aircok Inc., Seoul, Korea). The device was located at the center of each participant’s house where they spent most time. The data were sent to a server based on IoT throughout the study period. Before the analysis, data cleaning was performed. Zeroes, frozen concentration values for several hours and extreme peaks were removed from the dataset. We detected outliers using the mean (μ) and standard deviation (σ) normal distribution of the PM_2.5_ concentrations, set by μ ± 2.97 × σ, including 99.7% of the observations. To correct for possible errors in the light scattering methods, gravimetric measurements using a mini-volume air sampler (Model KMS-4100; KEMIK Corp., Seongnam, Korea) and an accurate aerosol spectrometer (11-D; Grimm, Ainring, Germany) were taken, respectively. Indoor PM_2.5_ concentrations reported by the IoT showed good linearity (R^2^ = 0.923) with the GRIMM reference and a moderate correlation (R^2^ = 0.451) with their co-located mini-volume air samplers. (Figure S1). Information about outdoor PM concentrations relating to the residential address was gathered from Air Korea, a national air pollution information system in South Korea (http://www.airkorea.or.kr).

### Statistical analysis

Data are shown as mean ± standard deviation for continuous variables and as number (%) for categorical variables. For non-continuous variables such as practice scores, an analysis of variance was used to confirm the difference between indoor and outdoor PM_2.5_ concentrations corresponding to the practice scores. In addition, data were compared using the t-test and variation analysis by classifying the frequency with which patients performed the practices into two categories: practiced or not practiced every day. Furthermore, logistic regression analysis was used to determine COPD exacerbations according to the difference between indoor and outdoor PM_2.5_ concentrations. The statistical significance level was set to *p* < 0.05, but 0.0125 was considered significant according to Bonferroni correction in the analysis of major outcomes of seasonal lifestyle practices, considering four seasons. All statistical analyses were performed using SPSS software (version 22, IBM Corp., Armonk, NY, USA).

## Results

### Baseline characteristics of the patients

A total of 110 patients with COPD were enrolled for the panel study. After excluding six patients due to missing data about indoor PM_2.5_ concentrations, 104 patients were finally enrolled for analysis. The mean age of patients was 67.4 ± 9.8 years, and 94 (90.4%) patients were male. Twenty-three (22.1%) patients were current smokers and 64 (61.5%) patients were ex-smokers with a mean of 33.7 ± 23.3 pack years. Among them, 38 (36.5%) patients had a history of acute exacerbations in the past year. All of them used inhalers. The mean COPD assessment test score was 17.0 ± 8.7. Dyspnea assessed with the modified Medical Research Council scale was relatively mild in a large proportion of patients. More than half (62, 59.6%) of patients had grade 1, but approximately 30% had grade 3 or higher (16 [15.4%] patients had grade 3 and 14 [13.5%] patients had grade 4) at enrollment. The mean total score of the COPD-specific version of the St. George’s Respiratory Questionnaire (SGRQ-C) was 38.43 ± 2.45 and mean R5 (resistance at 5 Hz)–R20 (resistance at 20 Hz) was 0.18 ± 0.03 cmH_2_O/l/s (Table 1).

**Table 1:** Baseline characteristics of the study participants.

Characteristics	N
Age, years (SD)	67.4 ± 9.8
Male, n (%)	94 (90.38)
Smoking status, n (%)	
Never smoker	17 (16.35)
Ex-smoker	64 (61.54)
Current-smoker	23 (22.12)
Smoking history (pack-year), mean (SD)	33.73 ± 23.33
mMRC, n (%)	
Grade 1	62 (59.62)
Grade 2	12 (11.54)
Grade 3	16 (15.38)
Grade 4	14 (13.46)
Acute exacerbation history last year	38 (36.54)
Inhaler usage, n (%)	
ICS + LABA	21 (20.19)
LAMA + LABA	33 (31.73)
LAMA + LABA + ICS	39 (37.5)
LABA + LAMA + SABA	3 (2.88)
ICS + LABA + LAMA + SABA	2 (1.92)
LAMA + SABA	1 (0.96)
LAMA	2 (1.92)
ICS + LAMA	1 (0.96)
Others	2 (1.92)
Lung function, mean (SD)	
PreBD FEV_1_, L (% predicted)	1.57 ± 0.54 (52.69 ± 17.11)
PreBD FVC, L (% predicted)	3.30 ± 0.84 (79.64 ± 16.27)
PostBD FEV_1_, L (% predicted)	1.62 ± 0.56 (54.53 ± 16.64)
PostBD FVC, L (% predicted)	3.28 ± 0.86 (80.52 ± 14.48)
CAT score, mean (SD)	17.04 ± 8.65
SGRQ total, mean (SD)	38.43 ± 2.45
R5-R20 (SD)	0.18 ± 0.03

*SD* standard deviation, *mMRC* Modified Medical Research Council, *ICS* inhaled corticosteroid, *LABA* long-acting beta 2 agonist, *BD* bronchodilator, *FEV*_*1*_ forced expiratory volume in one second, *FVC* forced vital capacity, *CAT* COPD assessment test.

*SGRQ* St. George’s Respiratory Questionnaire, *R5* resistance at 5 Hz, *R20* resistance at 20 Hz.

### PM_2.5_ concentrations and lifestyle practices to reduce PM exposure

**Figure 1:**
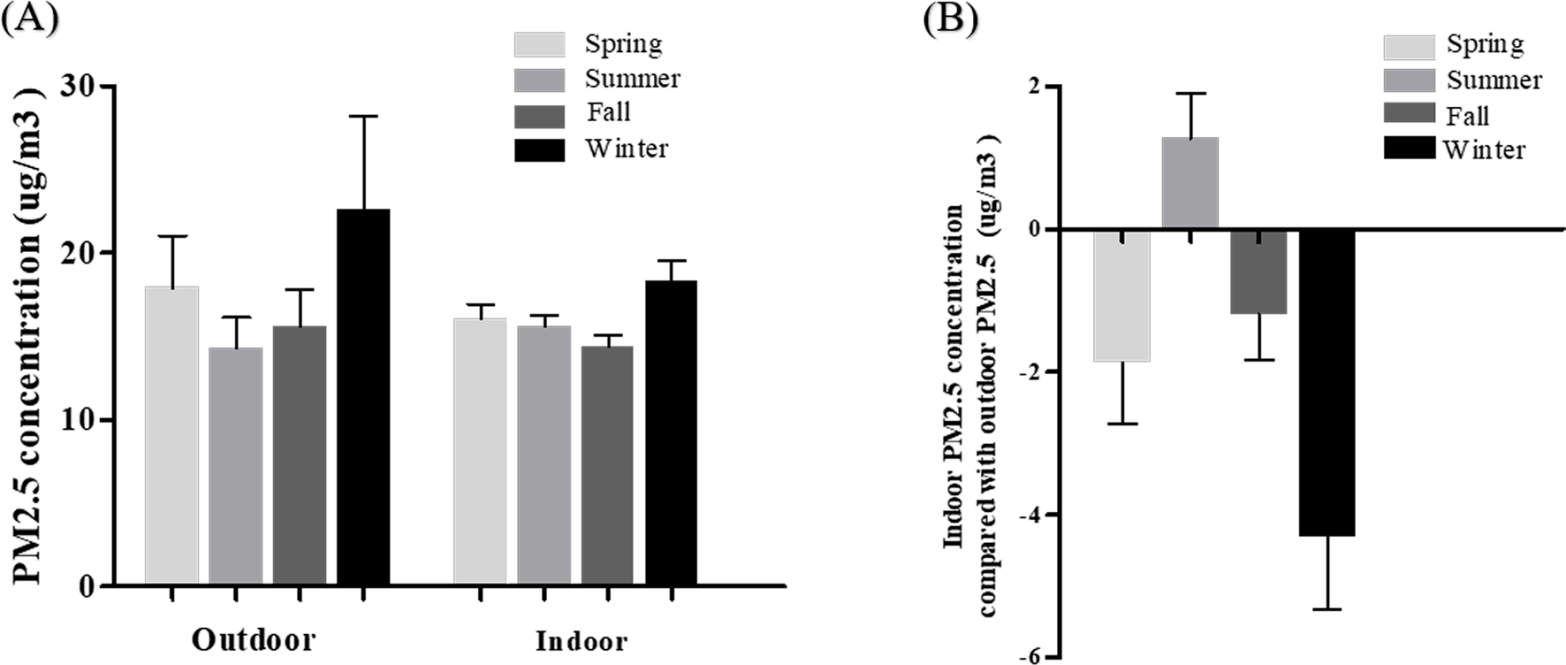
Mean PM_2.5_ concentrations by season. (**A**) Outdoor and indoor PM_2.5_ concentrations by season. (**B**) Indoor PM_2.5_ concentration compared with the outdoor PM_2.5_ concentration by season. The overall outdoor PM_2.5_ concentration was higher than the indoor PM_2.5_ concentration in all seasons except summer. The difference between indoor and outdoor PM_2.5_ concentrations was greatest in winter.

**Figure 2:**
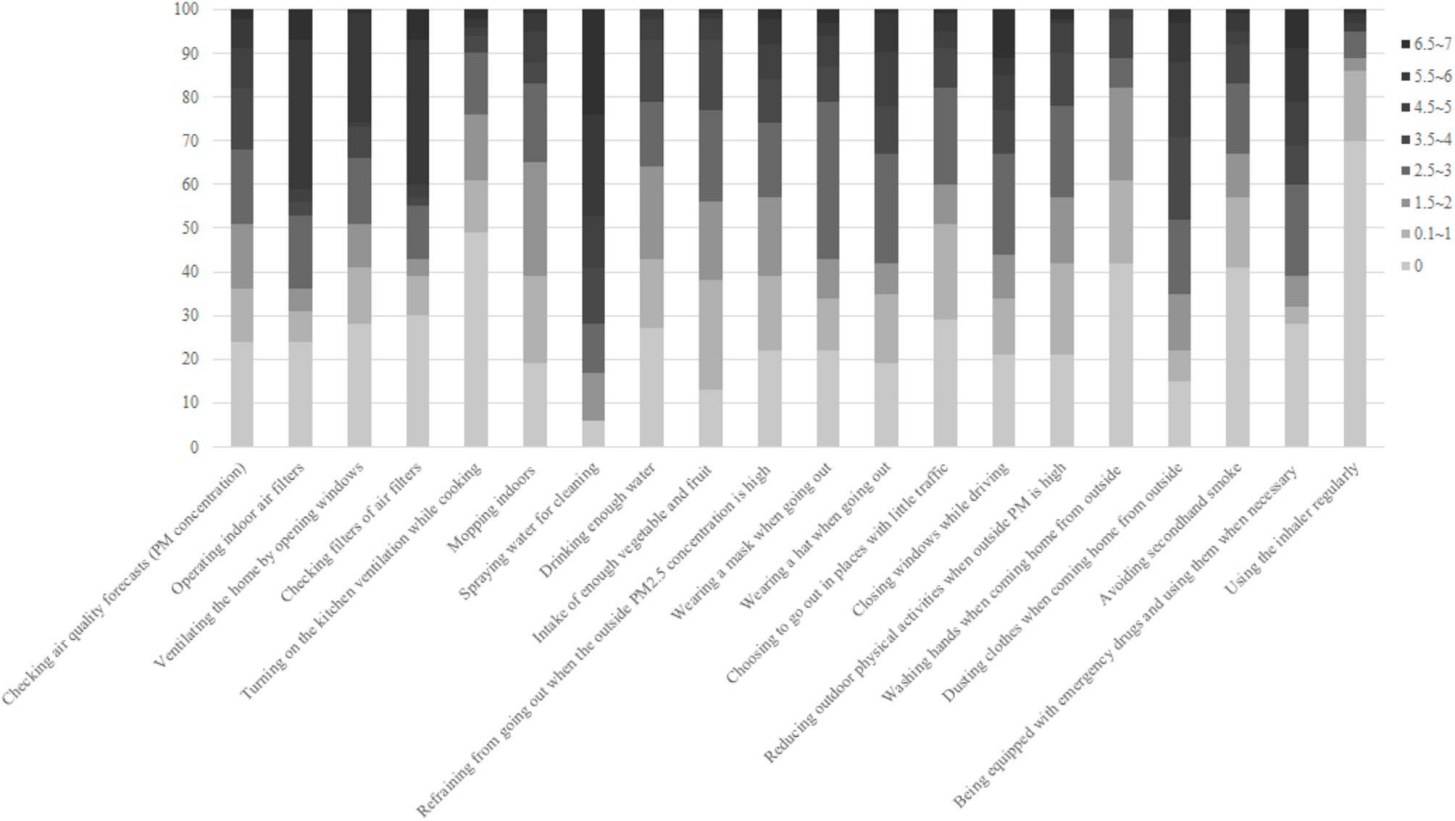
Lifestyle practices to avoid PM exposure. The 20 lifestyle items are presented according to the proportion (%) of patients. The questionnaire asked questions about how many days per week the patient usually performed the items. During the survey period, the questionnaire was completed twice, and the average scores are expressed.

PM_2.5_ concentrations were lower indoors than outdoors in all seasons except summer. The difference between indoor and outdoor PM_2.5_ concentrations was greatest in winter (− 4.31 ± 1.02 µg/m^3^), followed by spring (− 1.87 ± 0.85 µg/m^3^), fall (− 1.20 ± 0.63 µg/m^3^), and summer (+ 1.27 ± 0.63 µg/m^3^, Fig. 1). Among the 20 lifestyle items, those commonly practiced every day included turning on the kitchen ventilation while cooking (49.0%), washing hands after coming home (42.0%), and avoiding secondhand smoke (41.0%). On the contrary, items not commonly practiced were spraying water for cleaning (24.0%), closing windows while driving (11.0%), and being equipped with emergency drugs and using them when necessary (9.0%, Fig. 2).

**Figure 3:**
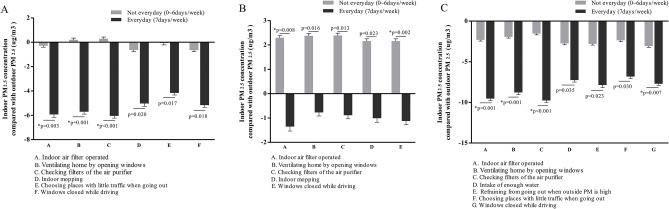
Indoor PM_2.5_ concentrations compared with outdoor PM_2.5_ concentrations over 1 year by season according to lifestyle practices to reduce PM exposure. (**A**) In spring, six lifestyle practices were correlated with a significant difference. (**B**) In summer, five lifestyle practices were correlated with a significant difference. (**C**) In winter, seven lifestyle practices were correlated with a significant difference.

**Figure 4:**
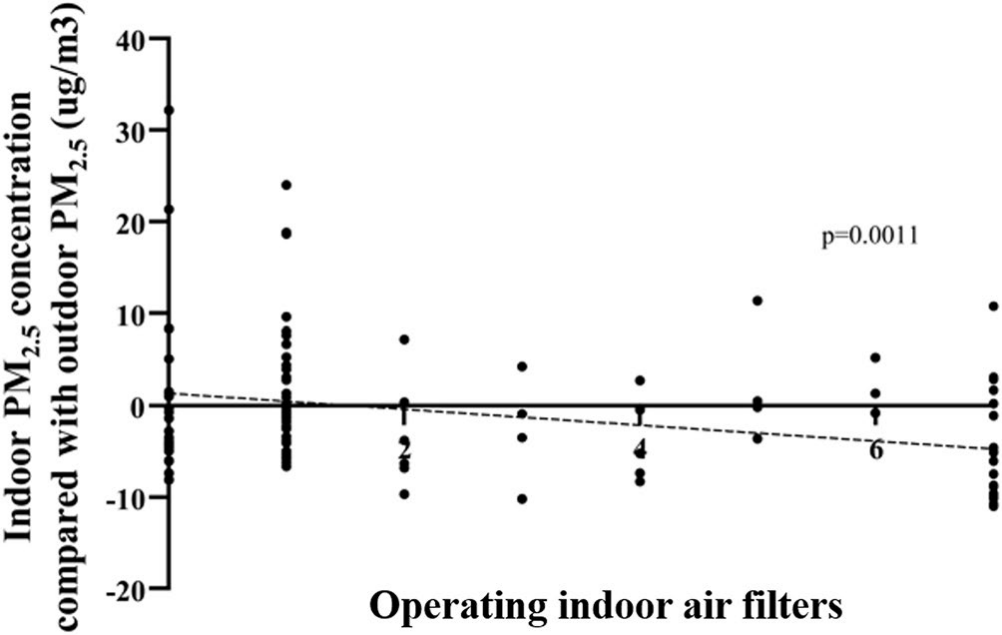
Indoor PM_2.5_ concentrations compared with outdoor PM_2.5_ concentrations according to the number of days of air filter use. The more days air filters were used, the better the indoor PM_2.5_ concentration was noted.

Some everyday lifestyle practices affected the difference between indoor and outdoor PM_2.5_ concentrations. The PM_2.5_ concentration was significantly lower indoors than outdoors in spring, summer, and winter when patients used indoor air filters, checked filters of air filters, ventilated the home by opening windows, and closed windows while driving with internal circulation mode. Other lifestyle practices that reduced the indoor PM_2.5_ concentration compared with the outdoor PM_2.5_ concentration in specific seasons included mopping indoors (spring and summer), choosing to go out in places with little traffic (spring and winter), and refraining from going out when the outside PM_2.5_ concentration was high (winter, Fig. 3). No significant association between the difference in indoor and outdoor PM_2.5_ concentrations and lifestyle practices was found in fall. Detailed seasonal differences are described in the online supplement. The more days air filters were used, the lower the indoor PM_2.5_ concentration was throughout the year (Fig. 4).

### Indoor and outdoor PM_2.5_ concentrations according to participants’ social environment

**Figure 5:**
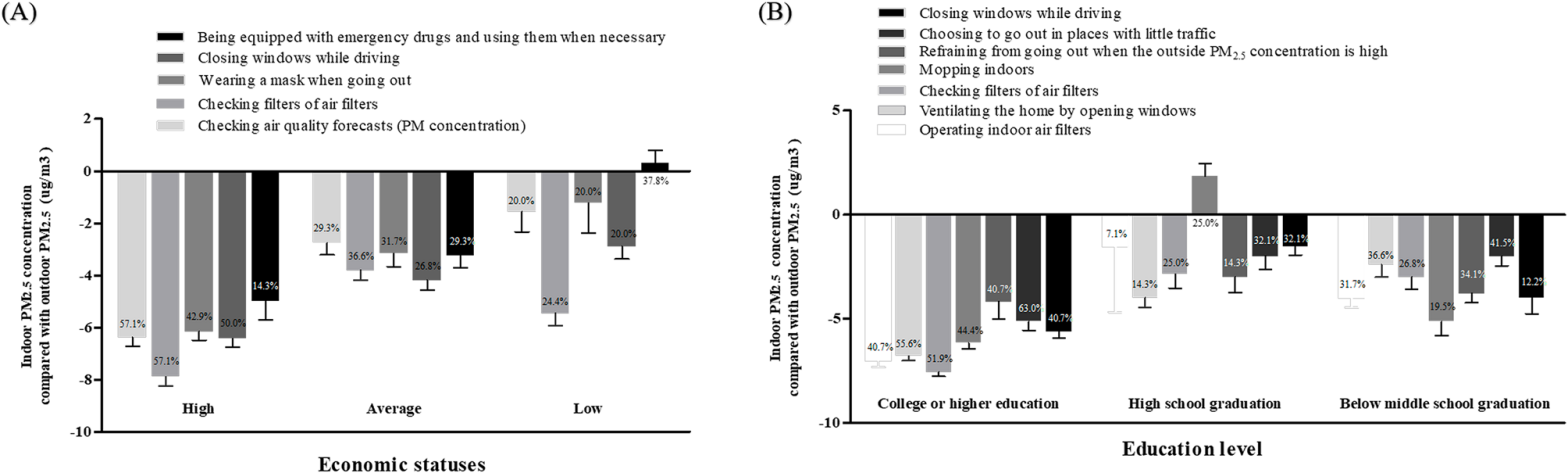
Indoor PM_2.5_ concentrations compared with outdoor PM_2.5_ concentrations according to the social background of patients. Differences were determined in terms of economic status (**A**) and educational level (**B**).

Further analysis was performed of participants’ social environment. The higher the economic status and educational level, the greater the difference between indoor and outdoor PM_2.5_ concentrations. When economic status was divided into three groups (high, middle, and low), the higher the economic status, the greater the difference between annual indoor and outdoor PM_2.5_ concentrations (high, − 4.71 ± 1.12 µg/m^3^; middle, + 0.17 ± 1.40 µg/m^3^; and low, − 1.93 ± 0.92 µg/m^3^; *p* = 0.086, Fig. 5A). These differences as economic levels were even more pronounced on some everyday lifestyle practices, including checking air quality forecasts (*p* = 0.012), checking filters of air filters (*p* = 0.023), wearing a mask when going out (*p* = 0.073), closing windows while driving with internal circulation mode (*p* = 0.042), and being equipped with emergency drugs and using them when necessary (*p* = 0.099, Fig. 5A). The difference between indoor and outdoor PM_2.5_ concentrations also varied according to the educational level, which was divided into three groups (higher than college graduation, high school graduation, and lower than middle school graduation). The higher the educational level, the greater the difference between annual indoor and outdoor PM_2.5_ concentrations (higher than college graduation, − 6.00 ± 1.14 µg/m^3^; high school graduation, − 1.98 ± 0.88 µg/m^3^; and less than middle school graduation, + 0.32 ± 1.40 µg/m^3^; *p* = 0.034, Fig. 5B). The difference between indoor and outdoor PM_2.5_ concentrations was more evident according to some everyday lifestyle practices including operating indoor air filters (*p* = 0.013), ventilating the home by opening windows (*p* = 0.005), checking filters of air filters (*p* = 0.055), mopping indoors (*p* = 0.075), refraining from going out when the outside PM_2.5_ concentration was high (*p* = 0.079), choosing to go out in places with little traffic (*p* = 0.061), and closing windows while driving with internal circulation mode (*p* = 0.021, Fig. 5B). Detailed differences are described in the online supplement.

### Relationship between indoor PM_2.5_ concentrations, lifestyle practices, and acute COPD exacerbations

**Figure 6:**
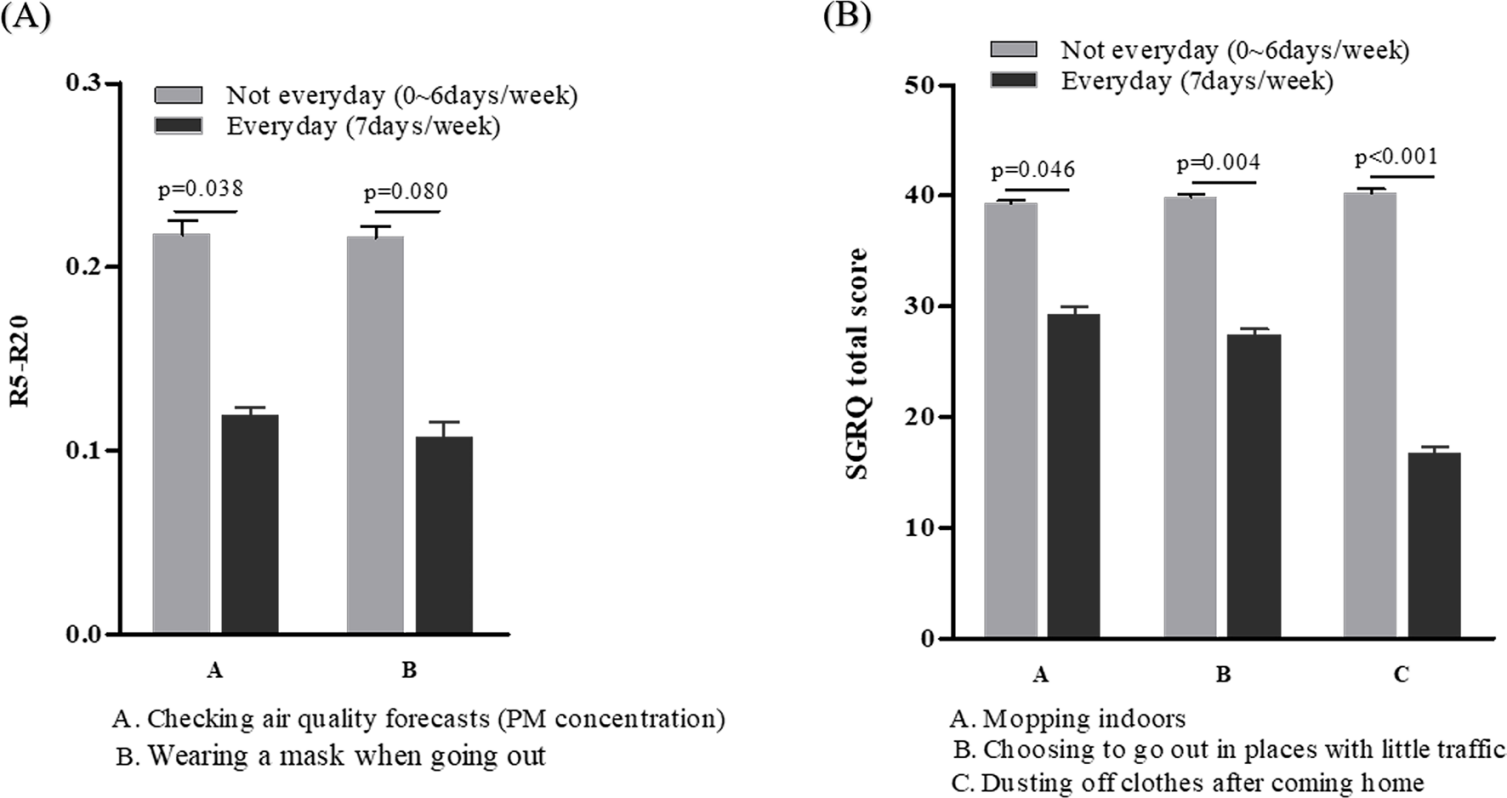
Relationship between COPD acute exacerbations and indoor/outdoor PM_2.5_ concentrations. (**A**) R5–R20 and lifestyle practices. Two lifestyle practices were correlated with a significant difference. (**B**) SGRQ-C scores and lifestyle practices.

R5–R20 was significantly lower for patients whose everyday lifestyle practices included checking air quality forecasts (0.12 ± 0.02 cmH_2_O/l/s, *p* = 0.038) and wearing a mask when going out (0.11 ± 0.03 cmH_2_O/l/s, *p* = 0.080, Fig. 6A). The SGRQ-C score was also lower for patients whose everyday lifestyle practices including mopping indoors (29.21 ± 4.29, *p* = 0.046), choosing to go out in places with little traffic (27.37 ± 3.17, *p* = 0.004), and dusting clothes when coming home from outside(16.73 ± 2.16, *p* < 0.001, Fig. 6B).

## Discussion

This study shows that PM_2.5_ concentrations can be affected by lifestyle practices and economic status according to the season. Some lifestyle practices were related with a significantly lower indoor PM_2.5_ concentration compared with the outdoor PM_2.5_ concentration in multiple seasons, including operating indoor air filters, ventilating the home by opening windows, checking filters of air filters, and closing windows while driving. Patients with a higher educational level or economic status had lower indoor PM_2.5_ concentrations. Lifestyle practices related to lower small airway resistance and SGRQ-C scores included checking air quality forecasts and mopping indoors. These results suggest that some lifestyle practices affect PM_2.5_ exposure and that exposure to PM_2.5_ can be reduced by adjusting these practices.

Some lifestyle practices were related with a lower indoor PM_2.5_ concentration compared with the outdoor PM_2.5_ concentration in all seasons except fall. Ventilating the home by opening windows was one such lifestyle practice. Many people wonder if regular ventilation through windows is helpful because outdoor PM_2.5_ concentrations are variable and sometimes higher than indoor PM_2.5_ concentrations. A previous study showed such ventilation is associated with reduced indoor PM_2.5_ concentrations even in cold seasons when the mean outdoor PM_2.5_ concentration is higher than that indoors^18^. This suggests that regular ventilation through windows does not adversely affect indoor PM_2.5_ concentrations if it is performed in conjunction with other lifestyle practices^19^. Many human activities can increase indoor PM_2.5_ concentrations such as walking, dressing, and cooking^20^. Even activities beneficial for indoor environments, such as sweeping, can dramatically increase indoor PM_2.5_ concentrations in the short term^21^. On the other hand, it would not work where the outdoor concentrations were twice as high as indoor ones^22^. All these findings suggests that indoor PM_2.5_ concentrations are variable and that regular ventilation through windows can be helpful depending outdoor conditions^21^. Meanwhile, the PM_2.5_ concentration in a vehicle becomes high if the windows are open when the outdoor PM_2.5_ concentration is high^23^. Our study found that closing windows while driving when the outdoor PM_2.5_ concentration was high improved the indoor PM_2.5_ concentration. Similarly, previous studies showed that driving with windows closed protects against traffic-related PM_2.5_ exposure^24,25^. Interestingly, this habit tended to be practiced more as the economic level of patients increased in our study.

Air filters are a well-studied intervention to reduce PM_2.5_ exposure. In a meta-analysis about air filter interventions for chronic respiratory diseases, air filters consistently improved indoor PM_2.5_ concentrations^26^. A recent randomized controlled trial showed that improvements of respiratory symptoms and acute exacerbations by an air filter invention were associated with reduced indoor PM_2.5_ concentrations^27^. A classroom-based air filter intervention significantly reduced PM_2.5_ and black carbon concentrations^28^. Use of indoor air filters significantly reduced indoor concentrations of PM_2.5_ and its components such as water soluble organics, NO_3_^–^, SO_4_^2–^, Zn^2+^, Pb^2+^, and K^+^^29^. In a study of children exposed to secondhand smoke, air filters not only reduced the level of airborne particles but also improved clinical outcomes such as unscheduled hospital visits due to asthma^30^. A short-term intervention operating indoor air filters improved various cardiovascular biomarkers in college students^31^. Everyday use of air filters is recommended to significantly improve indoor PM_2.5_ concentrations, and checking filters is also helpful^18,32^. In this study, everyday use of air filters reduced indoor PM_2.5_ concentrations.

Maintenance of a clean indoor environment is another important strategy to reduce indoor air pollution. Interestingly, indoor mopping was significantly associated with a reduced indoor PM_2.5_ concentration compared with the outdoor PM_2.5_ concentration in spring and summer. This is consistent with the previous recommendation for wet mopping to lower indoor pollution^33^. The previous finding that wet sweeping increases PM_2.5_ concentrations less than dry sweeping supports the recommendation for wet mopping^21^. Another noteworthy recommendation for the indoor environment is to use a vacuum with a high-efficiency particulate-absorbing (HEPA) filter. Use of a vacuum without an appropriate filter can stir up particles and deep dust, but use of a vacuum with a HEPA filter helps to control asthma by reducing dust exposure^34,35^.

In contrast with other seasons, we did not find any significant association between lifestyle practices and PM_2.5_ concentrations in fall. This can be explained by the finding that the difference between indoor and outdoor PM_2.5_ concentrations was smallest in this season. A national database also showed that the mean outdoor PM_2.5_ concentration is lower in fall than in other seasons^36^. By contrast, relatively strong associations were found in winter, during which the difference between indoor and outdoor PM_2.5_ concentration was largest. There is evidence of seasonal variation in the rate of hospital admissions for COPD, with more exacerbations occurring during winter^37,38^. Exacerbations are also associated with cooler temperatures^39,40^. This suggests that lifestyle correction can reduce the risk of COPD more effectively in winter, when patients are usually more vulnerable.

Some other lifestyle practices, such as refraining from going out when the outside PM_2.5_ concentration was high and choosing to go out in places with little traffic, were also effective, which might not be directly related with indoor PM_2.5_ concentrations. Consistently, a previous study showed that reducing outdoor physical activities and staying inside on days when outdoor PM concentrations are very high improves indoor PM_2.5_ concentrations^32^. The U.S. Air Quality Index recommends to stay indoors in an area with filtered air and to avoid outdoor activities when the outdoor PM concentration is high. With these lifestyle practices, people can refrain from breathing rapidly and deeply, which increases PM_2.5_ inhalation^33^. This recommendation also applies to COPD patients^41^. It is unclear how these lifestyle practices also have beneficial effects on indoor PM_2.5_ concentrations. It may be because people who perform these lifestyle practices also perform other lifestyles that have significantly beneficial effects on indoor PM_2.5_ concentrations. In our study, participants who chose to go out in places with little traffic also performed other effective lifestyle practices (Figure S3). A group of participants may perform several lifestyle practices simultaneously to reduce PM exposure.

Long-term and constant exposure to high PM_2.5_ concentrations leads to aggravation of respiratory symptoms and acute exacerbations of COPD via inflammation, oxidative stress, immune dysfunction, and alterations of the airway epithelial structure and microbiome^42,43^. Therefore, lowering exposure to higher PM_2.5_ concentrations is a viable approach to reduce acute exacerbations, attenuate COPD progression, and decrease the associated healthcare burden^42^. In our study, we evaluated whether clinical outcomes were improved by lifestyle practices that reduced PM_2.5_ concentrations. Checking air quality forecasts (PM_2.5_ concentrations) daily and wearing a mask when going out correlated with lower R5–R20 levels. In addition, mopping indoors, choosing to go out in places with little traffic, and dusting off clothes after coming home were also associated with lower SGRQ-C total scores. Considering the aforementioned findings, specific lifestyle practices can improve clinical outcomes of COPD patients by reducing PM_2.5_ exposure.

Several limitations can be suggested in this study. First, the lifestyle questionnaire was based on people’s memories, which can sometimes be biased. This may be why only lifestyle practices performed every day (7 days/week) made a significant difference. Second, the PM_2.5_ concentrations from IoT-based sensors showed a moderate correlation with their co-located mini-volume air samplers. In this study, the correlation of IoT with the Grimm reference was excellent (R^2^ = 0.923), but its mean concentrations showed a relatively lower correlation with 24-h volume sampling. This is likely due to different conditions at each deployment site, such as chemical PM_2.5_ compositions, temperature, relative humidity, and indoor sources. Therefore, it is crucial to develop more accurate real-time monitoring of PM_2.5_ concentrations since gravimetric monitoring is impractical for long-term home-based cohorts due to noise and device size. Despite this limitation, the findings of the current study are important for several reasons. First, PM has a significant impact on development and progression of COPD, but research about individual management for PM_2.5_ exposure is lacking. This study reported the detailed lifestyle practices of COPD patients and how they can affect PM_2.5_ concentrations. It also found a relationship between PM_2.5_ concentrations and acute COPD exacerbations. Second, IoT-based PM_2.5_-measuring sensors were installed in patients’ home, and indoor PM concentrations were monitored continuously. This study provides evidence with more accurate data. Third, this study collected data over 1 year and noted dynamic changes in PM_2.5_ concentrations according to the season. Using this study design, the effect of each lifestyle practice in each season could be determined.

In conclusion, lifestyle practices are associated with indoor PM_2.5_ concentrations and can even affect clinical outcomes, including small airway resistance and quality of life of COPD patients. Some lifestyle practices such as operating air filters and ventilating the home by opening windows help to reduce PM_2.5_ concentrations, and these can be included as scientific guidance to reduce exposure of patients with COPD to PM_2.5_.

## Supplementary Information

Supplementary Information 1.

## Data Availability

The datasets used and/or analysed during the current study are available from the corresponding author on reasonable request.

## Abbreviations

COPD: Chronic obstructive pulmonary disease
FEV_1_: Forced expiratory volume in 1 s
FVC: Forced vital capacity
HEPA: High-efficiency particulate-absorbing
IoT: Internet of Things
PM: Particulate matter
PM_2.5_: Particulate matter with a diameter smaller than 2.5 µm
SGRQ-C: COPD-specific version of the St. George’s Respiratory Questionnaire
