# Supplementary material for: Lifestyle practices that reduce seasonal PM2.5 exposure and their impact on COPD
Source: Sci Rep. 2023 Jul 21;13:11822. doi: 10.1038/s41598-023-38714-5 (PMC10361977; doi:10.1038/s41598-023-38714-5)

**Supplementary material**

**Lifestyle practices that reduce seasonal PM_2.5_ exposure and their impact on COPD**

Hajeong Kim^1,6^, Jin-Young Huh^1,7^, Geunjoo Na^2^, Shinhee Park^3,8^, Seung Won Ra^4^, Sung-Yoon Kang^5^, Ho Cheol Kim^1^, Hwan Cheol Kim^2*^, and Sei Won Lee^1*^

^1^Department of Pulmonary and Critical Care Medicine, University of Ulsan College of Medicine, Asan Medical Center, Seoul, Republic of Korea

^2^Department of Occupational and Environmental Medicine, College of Medicine, Inha University, Incheon, Republic of Korea

^3^Department of Pulmonary, Allergy and Critical Care Medicine, Gangneung Asan Hospital, Gangneung, Republic of Korea

^4^Division of Pulmonary and Critical Care Medicine, Department of Internal Medicine, Ulsan University Hospital, University of Ulsan College of Medicine, Ulsan, Republic of Korea

^5^Department of Internal Medicine, Gachon University Gil Medical Center, Incheon, Republic of Korea

^6^Division of Pulmonary, Allergy and Critical Care Medicine, Department of Internal Medicine, Hallym University Kangdong Sacred Heart Hospital, Seoul, Republic of Korea

^7^Division of Pulmonary, Allergy and Critical Care Medicine, Department of Internal Medicine, Chung-Ang University Gwangmyeong Hospital, Chung-Ang University College of Medicine, Gwangmyeong, Republic of Korea

^8^Division of Allergy and Respiratory Medicine, Department of Internal Medicine, Soonchunhyang University Bucheon Hospital, Bucheon, South Korea

*These authors contributed equally.

**Table S1**. Questionnaire about lifestyle practices to reduce PM exposure

| **Practice Item** | **Frequency**  Never practiced Practiced everyday  🡨 🡪 | | | | | | | |
| --- | --- | --- | --- | --- | --- | --- | --- | --- |
| 1. I frequently checked the forecast for fine dust and changes in its concentration. | (0) | (1) | (2) | (3) | (4) | (5) | (6) | (7) |
| 1. I kept air purifiers on (if there was no air purifier, please choose the score (0)). | (0) | (1) | (2) | (3) | (4) | (5) | (6) | (7) |
| 1. While the air purifier was on, I also regularly ventilated the room by opening windows (if there was no air purifier, please choose the score (0)). | (0) | (1) | (2) | (3) | (4) | (5) | (6) | (7) |
| 1. While the air purifier was on, I also regularly checked and replaced the filters (if there was no air purifier, please choose the score (0)). | (0) | (1) | (2) | (3) | (4) | (5) | (6) | (7) |
| 1. I turned on the kitchen ventilator (fan) while cooking and made sure that the room was ventilated afterward. | (0) | (1) | (2) | (3) | (4) | (5) | (6) | (7) |
| 1. When cleaning indoors, I mopped instead of using a vacuum cleaner. | (0) | (1) | (2) | (3) | (4) | (5) | (6) | (7) |
| 1. When cleaning indoors, I sprayed water before mopping. | (0) | (1) | (2) | (3) | (4) | (5) | (6) | (7) |
| 1. I drank plenty of water to remove waste products from my body. | (0) | (1) | (2) | (3) | (4) | (5) | (6) | (7) |
| 1. I ate a lot of vegetables and fruits with antioxidant properties. | (0) | (1) | (2) | (3) | (4) | (5) | (6) | (7) |
| 1. I limited the frequency at which I went out or did outdoor activities. | (0) | (1) | (2) | (3) | (4) | (5) | (6) | (7) |
| 1. I wore a health mask (FDA-certified) properly when I went out. | (0) | (1) | (2) | (3) | (4) | (5) | (6) | (7) |
| 1. I minimized exposure of my body by wearing a hat, long sleeves, safety glasses, etc. | (0) | (1) | (2) | (3) | (4) | (5) | (6) | (7) |
| 1. I refrained from going to places with high concentrations of fine dust, such as areas with heavy traffic, factories, etc., as much as possible. | (0) | (1) | (2) | (3) | (4) | (5) | (6) | (7) |
| 1. I switched to indoor circulation mode with the windows closed while driving. | (0) | (1) | (2) | (3) | (4) | (5) | (6) | (7) |
| 1. I reduced the duration or intensity of outdoor physical activities (for example, I chose to walk instead of run). | (0) | (1) | (2) | (3) | (4) | (5) | (6) | (7) |
| 1. I washed myself after coming home (washing all over, especially hands, feet, eyes, and nose with running water, and brushing teeth). | (0) | (1) | (2) | (3) | (4) | (5) | (6) | (7) |
| 1. I dusted off my clothes before entering the house. | (0) | (1) | (2) | (3) | (4) | (5) | (6) | (7) |
| 1. I avoided secondhand smoke (in the case of a non-smoker)/I refrained from smoking (in the case of a smoker). | (0) | (1) | (2) | (3) | (4) | (5) | (6) | (7) |
| 1. I purchased an emergency inhaler for symptom exacerbations and used it when necessary. | (0) | (1) | (2) | (3) | (4) | (5) | (6) | (7) |
| 1. I used an inhaler regularly in the instructed way. | (0) | (1) | (2) | (3) | (4) | (5) | (6) | (7) |

The questionnaire started with “On a scale from 0 (never practiced) to 7 (practiced every day), how many days have you usually practiced the following items when the concentration of particulate matter was high (≥35 µg/m^3^) or very high (≥75 µg/m^3^) in the last year?”

**Table S2**. Indoor PM_2.5_ concentration (µg/m^3^) compared with the outdoor PM_2.5_ concentration in December 2019 according to whether lifestyle practices to reduce PM exposure were performed every day

|  | Not performed every day (0–6 days/week) | Performed every day  (7 days/week) | p value |
| --- | --- | --- | --- |
| Turning on kitchen ventilation while cooking | –8.46±1.24 | –11.52±1.06 | 0.066 |
| Mopping indoors | –10.38±1.05 | –10.20±1.31 | 0.917 |
| Spraying water for cleaning | –10.31±0.89 | –10.28±2.14 | 0.989 |
| Drinking enough water | –9.82±1.08 | –11.41±1.21 | 0.364 |
| Eating enough vegetables and fruits | –10.41±0.98 | –10.25±1.52 | 0.925 |
| Wearing a hat when going out | –9.82±0.94 | –11.79±1.61 | 0.296 |
| Choosing to go out in places with little traffic | –9.53±1.02 | –11.47±1.34 | 0.244 |
| Closing windows while driving | –11.14±1.02 | –8.67±1.34 | 0.152 |
| Reducing outdoor physical activities when the outside PM_2.5_ concentration is high | –10.06±0.95 | –10.77±1.52 | 0.676 |
| Washing hands after coming home | –9.69±1.26 | –10.91±1.05 | 0.458 |
| Dusting off clothes after coming home | –10.09±0.95 | –11.0±1.62 | 0.631 |
| Avoiding secondhand smoke | –10.31±1.05 | –10.31±1.27 | >0.999 |
| Being equipped with emergency drugs and using them when necessary | –10.12±1.05 | –10.79±1.33 | 0.699 |
| Using an inhaler regularly | –8.35±2.00 | –10.91±0.89 | 0.203 |

Data are presented as mean ± standard error.

**Figure legends**

**Figure S1**. The relationship of IoT senor-based light scattering measurement with (A) an aerosol spectrometer reference (11-D; Grimm) and (B) the gravimetric mini-volume air sampler (KMS-4100; KEMIK Corp.,) data for indoor PM_2.5_ concentrations. The real-time correlation of light scattering measurements, and 24 h averages of indoor PM_2.5_ concentrations from the IoT sensors and their co-located mini-volume air samplers were analyzed. The slope and R^2^ value were calculated by least squares regression.

**
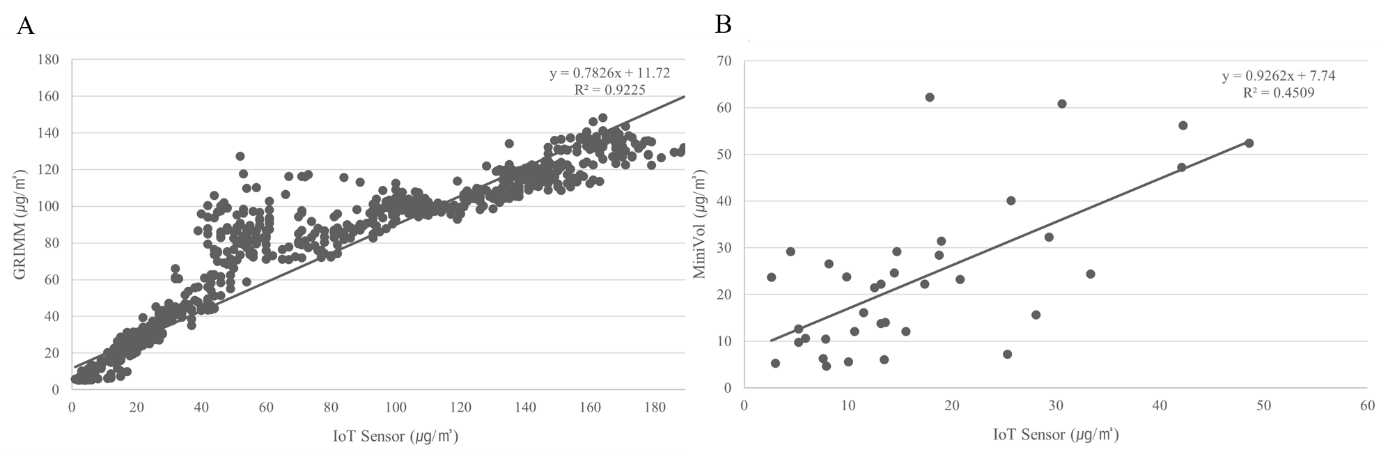
**

**Figure S2**. Lifestyle practices to avoid PM exposure. Distribution of patients that performed lifestyle practices that help reduce indoor PM concentrations.

(A) Questionnaire completed at enrollment.


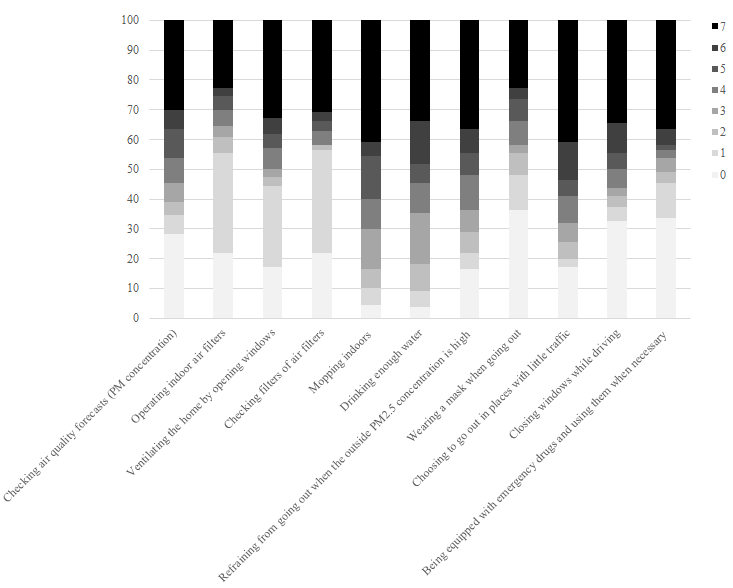


(B) Questionnaire completed at the end of the study.


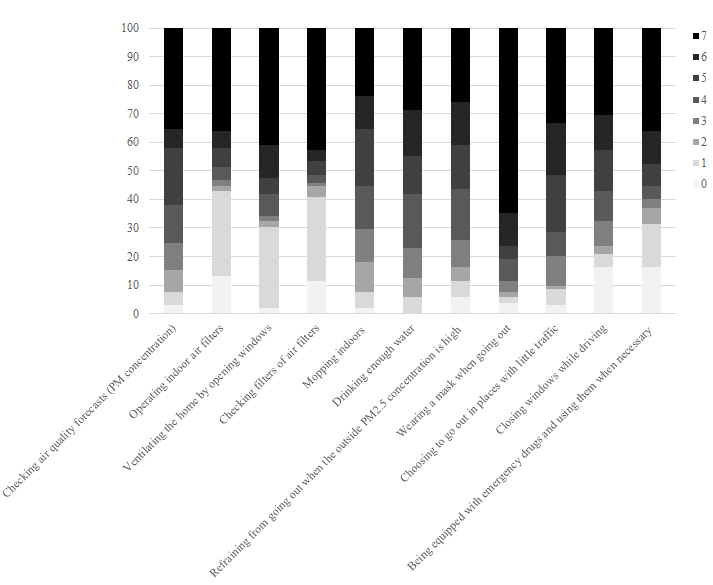


**Figure S3**. Correlations between lifestyle practices to reduce PM exposure


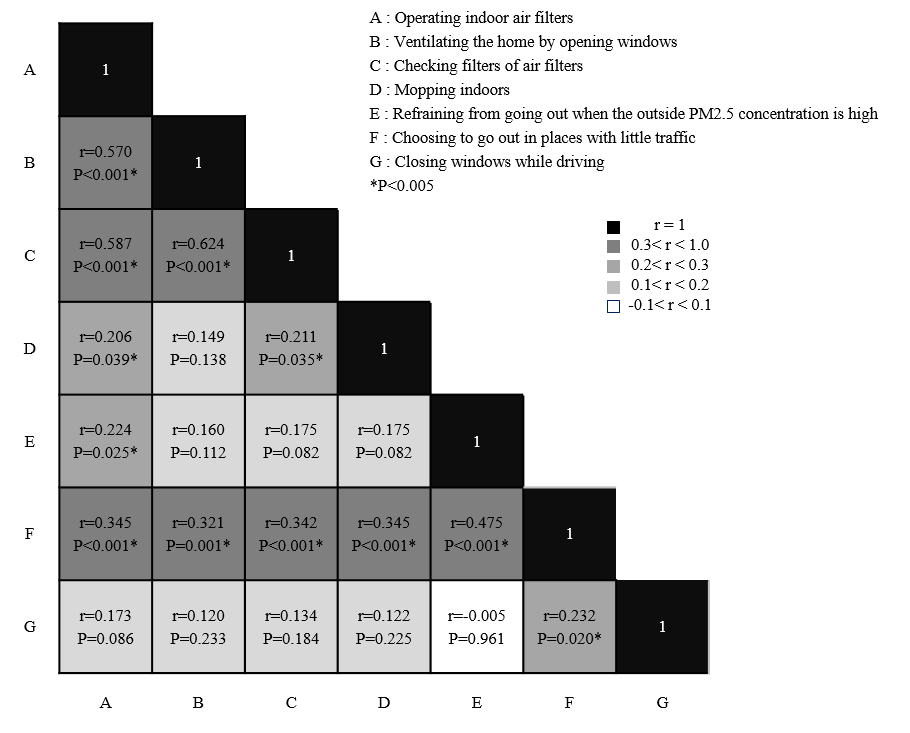

Supplement: Supplementary file 1 — Supplementary Information 1. [file 41598_2023_38714_MOESM1_ESM.docx]
